# Supplementary material for: Short antisense oligonucleotides alleviate the pleiotropic toxicity of RNA harboring expanded CGG repeats
Source: Nat Commun. 2021 Feb 24;12:1265. doi: 10.1038/s41467-021-21021-w (PMC7904788; doi:10.1038/s41467-021-21021-w)
Supplement: Supplementary file 1 — Reporting Summary [file 41467_2021_21021_MOESM1_ESM.pdf]

nature research

Corresponding author(s): Krzysztof Sobczak

Last updated by author(s): Dec 16, 2020

Reporting Summary

Nature Research wishes to improve the reproducibility of the work that we publish. This form provides structure for consistency and transparency in reporting. For further information on Nature Research policies, see Authors & Referees and the Editorial Policy Checklist .

Statistics

For all statistical analyses, confirm that the following items are present in the figure legend, table legend, main text, or Methods section.

n/a	Confirmed	
☐	☒	The exact sample size (*n*) for each experimental group/condition, given as a discrete number and unit of measurement
☐	☒	A statement on whether measurements were taken from distinct samples or whether the same sample was measured repeatedly
☐	☒	The statistical test(s) used AND whether they are one- or two-sided<br>*Only common tests should be described solely by name; describe more complex techniques in the Methods section.*
☐	☒	A description of all covariates tested
☐	☒	A description of any assumptions or corrections, such as tests of normality and adjustment for multiple comparisons
☐	☒	A full description of the statistical parameters including central tendency (e.g. means) or other basic estimates (e.g. regression coefficient) AND variation (e.g. standard deviation) or associated estimates of uncertainty (e.g. confidence intervals)
☐	☒	For null hypothesis testing, the test statistic (e.g. *F*, *t*, *r*) with confidence intervals, effect sizes, degrees of freedom and *P* value noted<br>*Give P values as exact values whenever suitable.*
☒	☐	For Bayesian analysis, information on the choice of priors and Markov chain Monte Carlo settings
☒	☐	For hierarchical and complex designs, identification of the appropriate level for tests and full reporting of outcomes
☐	☒	Estimates of effect sizes (e.g. Cohen's *d*, Pearson's *r*), indicating how they were calculated

Our web collection on statistics for biologists contains articles on many of the points above.

Software and code

Policy information about availability of computer code

Data collection	Flow cytometry data were collected and analyzed using guavaSoft™ software, version 3.1.1 (Merck).
Data analysis	Analysis of gels was performed using Multi Gauge 3.0 software (FujiFilm), GeneTools 4.02 (Syngene) and ImageQuant TL 8.1.0.0 (Cytiva). Microscopic images were analyzed using ImageJ 1.51j8 and Fiji 2.0.0-rc-69/1.52p software. RNA-seq data were analyzed using Illumina bcl2fastq (2.19), Skewer (version 0.2.2)52, Trimmomatic 0.3953, HISAT2 2.1.0 aligner54, FastQC 0.11.5 software (http://www.bioinformatics.babraham.ac.uk/projects/fastqc/), STAR 2.7.1a algorithm55, Qualimap 2.2.2 software56, RSEM 1.3.0, R 3.3.1, DESeq2 package, voom+limma pipeline, PANTHER14.1,. Statistical analysis was performed using Statistica software version 10, Prism software version 7 or Microsoft Excel 2010 version 14.0.7237.5000 and 2019 version 1808.

For manuscripts utilizing custom algorithms or software that are central to the research but not yet described in published literature, software must be made available to editors/reviewers. We strongly encourage code deposition in a community repository (e.g. GitHub). See the Nature Research guidelines for submitting code & software for further information.

Data

Policy information about availability of data

All manuscripts must include a data availability statement. This statement should provide the following information, where applicable:

- Accession codes, unique identifiers, or web links for publicly available datasets
- A list of figures that have associated raw data
- A description of any restrictions on data availability

RNA-seq data generated and analyzed during the current study are available in the NCBI SRA database. SRA accession: PRJNA577423. Publicly available datasets from https://www.ensembl.org/ were used in this study. The source data underlying Figs 1b-f, 2b–e, 3a-e, 4c-f, 5c and - d e and Supplementary Figs S1c-g, S2b-e, S3a-e, S4a and b, S6a and c-e f are provided with this paper as a Source Data file. The source data underlying Fig. 5a are provided in Supplementary Table Data S1 and S2. The source data underlying Fig. 5b and S6b are provided in Supplementary Table Data S3 and S4, respectively. The code used to generate heatmap presented in Supplementary Fig. S5 is available in the GitHub repository, https://github.com/MagdalenaDerbis/R-script-to-generate-heatmap/blob/main/

Supplementary%20R%20script%20to%20generate%20heatmap.R. Other data that support the findings of this study are available from the corresponding author upon reasonable request.

Field-specific reporting

Please select the one below that is the best fit for your research. If you are not sure, read the appropriate sections before making your selection.

- [x] Life sciences
- [ ] Behavioural & social sciences
- [ ] Ecological, evolutionary & environmental sciences

For a reference copy of the document with all sections, see nature.com/documents/nr-reporting-summary-flat.pdf

Life sciences study design

All studies must disclose on these points even when the disclosure is negative.

Sample size

For most cases, biological triplicate experiments were performed unless otherwise noted. Details on sample size for experiments (N) were indicated in methods and figure legends. No statistical methods were used to predetermine the sample size. Sample sizes for experiments were estimated based on previous experience with similar setups that showed significance. Wojtkowiak-Szlachcic, A. et al. Short antisense-locked nucleic acids (all-LNAs) correct alternative splicing abnormalities in myotonic dystrophy. Nucleic Acids Res. 43, 3318–3331 (2015); Nakamori, M., Taylor, K., Mochizuki, H., Sobczak, K. & Takahashi, M. P. Oral administration of erythromycin decreases RNA toxicity in myotonic dystrophy. Ann. Clin. Transl. Neurol. 3, 42–54 (2016). De Winter, J. C. F. Using the Student's t-test with extremely small sample sizes. Pract. Assessment, Res. Eval. Pract. Assess. 18, 10 (2013).
The sample size of the animal experiments has been established based on "resource equation method" as described in Festing, M. F. W. & Altman, D. G. Guidelines for the Design and Statistical Analysis of Experiments Using Laboratory Animals. ILAR J. 43, 244–258 (2002) and in Charan, J. & Kantharia, N. How to calculate sample size in animal studies? J. Pharmacol. Pharmacother. 4, 303–306 (2013). The E-value was set to 10, which results in the smallest possible number of animal use that is within the tresholds of this method. This E-value translates into a total number of 12 mice for an experiment consisted of 2 groups. This sample size was then adjusted for expected attrition to accommodate potential mortality due to surgical procedures. The final corrected sample size was therefore calculated as 14 mice.

Data exclusions

The only data exclusions concerned RNA-seq data analysis. Exlusion criteria were not pre-established. Single gene was excluded form Pearson correlation analysis and single gene was excluded from heatmap creation because of their outlier characteristics (they were indicated in methods and figure legends). No other data were excluded form analysis.

Replication

Cellular and in vitro experiments were repeated independently at least two times with similar results.

Randomization

For quantification of nuclei from microscopic images, random images of specified size generated from whole photomicrographs were used. Animals were randomized into different treatment groups.

Blinding

Specified number of nuclei was counted per mice form microscopic images by a researcher who was blind to the treatment groups.

Reporting for specific materials, systems and methods

We require information from authors about some types of materials, experimental systems and methods used in many studies. Here, indicate whether each material, system or method listed is relevant to your study. If you are not sure if a list item applies to your research, read the appropriate section before selecting a response.

Materials & experimental systems

n/a	Involved in the study	
☐	☒	Antibodies
☐	☒	Eukaryotic cell lines
☒	☐	Palaeontology
☐	☒	Animals and other organisms
☒	☐	Human research participants
☒	☐	Clinical data

Methods

n/a	Involved in the study	
☒	☐	ChIP-seq
☐	☒	Flow cytometry
☒	☐	MRI-based neuroimaging

Antibodies

Antibodies used

rabbit anti-FMRP (ab17722, Abcam)
rabbit anti-alpha-tubulin (ab52866, Abcam)
rabbit anti-QKI (ab126742, Abcam)
rabbit anti-SLC40A1 (NBP1-21502, Novus Biologicals)
rabbit anti-VKORC1L1 (PA5-48618, Thermo Fisher Scientific)
rabbit anti-GFAP (NB300-141, Novus Biologicals)
rabbit anti-TNF-alpha (ab66579, Abcam)
rabbit anti-CD68 (ab125212, Abcam)

rabbit anti-AIF1 (019-19741, Wako)
rabbit anti-KANSL1L (NBP2-14139, Novus Biologicals)
rabbit anti-DAZAP1 (PA5-41887, Thermo Fisher Scientific)
rat anti- PPP1R1B (MAB4230, R&D Systems)
mouse anti-alpha-tubulin (T6199, Merck)
mouse anti-GAPDH (sc-47724, Santa Cruz Biotechnology)
mouse anti-FMRpolyG 8FM (described in Buijsen, R. A. M. et al. FMRpolyG-positive inclusions in CNS and non-CNS organs of a fragile X premutation carrier with fragile X-associated tremor/ataxia syndrome. Acta Neuropathol. Commun. 2, 1–5 (2014))
anti-rabbit (AS09 602, Agrisera)
anti-rabbit (A9169, Merck)
anti-rabbit IRDye 800CW (926-32211, Li-COR)
anti-rat (712-025-153, JIR)
anti-mouse (sc-2005, Santa Cruz Biotechnology)
anti-mouse (A9044, Merck)
anti-mouse IRDye 680RD (926-68070, Li-COR)
anti-mouse (BMK-2202, Vector Labs)

Validation

The 8FM antibody has been described and validated on human tissue by Buijsen et al. 2014. Hukema et al. 2015 and Castro et al. 2017 further employed the 8FM antibody on the P90CGG mouse model. For the current study, prior to formal experiments, 8FM stained cerebellum tissue sections from P90CGG.DOX+ mice were compared to the 8FM stained tissue sections of age-matched P90CGG.DOX- mice and 90CGG.DOX+ mice for validation purposes. 8FM+ Intranuclear inclusions have only been identified in the sections obtained from P90CGG.DOX+ mice. Buijsen, R. A. M. et al. FMRpolyG-positive inclusions in CNS and non-CNS organs of a fragile X premutation carrier with fragile X-associated tremor/ataxia syndrome. Acta Neuropathol. Commun. 2, 1–5 (2014). Castro, H. et al. Selective rescue of heightened anxiety but not gait ataxia in a premutation 90CGG mouse model of Fragile X-associated tremor/ataxia syndrome. Hum. Mol. Genet. 26, 2133–2145 (2017). All other antibodies were validated by staining positive cell lines/tissues using protocols provided from manufactory before doing formal experiments. The specificity was determined by the detection of the product of the expected size according to manufacturer's protocols and published data.

Eukaryotic cell lines

Policy information about cell lines

Cell line source(s)

COS7 cell line was purchased form ATCC. Fibroblasts were obtained from Anita Bhattacharyya (Waisman Center, University of Wisconsin-Madison, Madison, USA) - C0603, FX11-02 and WC26 and from Paul J. Hagerman (MIND Institute, UC Davis Health, University of California, Davis, USA) - C6 and F3. GM23963 and GM04033 cell lines were purchased from Corieli Institute.

Authentication

Fibroblasts were authenticated by providers as described in Rovozzo, R. et al. CGG repeats in the 5'UTR of FMR1 RNA regulate translation of other RNAs localized in the same RNA granules. PLoS One 11, (2016) and Garcia-Arocena, D. et al. Fibroblast phenotype in male carriers of FMR1 premutation alleles. Hum. Mol. Genet. 19, (2009). The length of CGG repeats for C0603 cell line was verified by CGG Repeat Primed PCR followed by capillary electrophoresis. The 1073-07 cell line was verified via Sanger sequencing. No additional authentication was performed for this and other cell lines.

Mycoplasma contamination

Cell lines were not tested for mycoplasma contamination.

Commonly misidentified lines
(See ICLAC register)

No misidenified cell lines were used in the study.

Animals and other organisms

Policy information about studies involving animals; ARRIVE guidelines recommended for reporting animal research

Laboratory animals

Inducible transgenic P90CGG mouse was obtained as described in Hukema, R. K. et al. Reversibility of neuropathology and motor deficits in an inducible mouse model for FXTAS. Hum. Mol. Genet. 24, 4948–4957 (2015). Sex - male, age - 4 weeks at the beginning of experiment, 17-18 weeks at the end. All mice were bred and maintained in the animal facility of the Institute of Biology, Otto von Guericke University Magdeburg, Germany under standard laboratory conditions. The facility was kept on an inverted 12-hour dark/light cycle (lights off at 7 am with 30 minutes of dim phase), with regulated room temperature (21°C) and humidity (50-60%). Mice were weaned four weeks after birth and provided with ad libitum food (R/M-H V- 1534 or R/M-H-A153-D04004 +4600 mg/kg doxycycline hyclate, ssniff) and water.

Wild animals

The study did not involve wild animals.

Field-collected samples

The study did not involve samples collected from the field.

Ethics oversight

All experimental procedures were approved by local ethics committee Landesverwaltungsamt Sachsen-Anhalt (CEEA# 42502-2-1219UniMD) and met the guidlines of local and European regulations (European Union directive no. 2010/63/EU).

Note that full information on the approval of the study protocol must also be provided in the manuscript.

Flow Cytometry

Plots

Confirm that:

- [x] The axis labels state the marker and fluorochrome used (e.g. CD4-FITC).
- [x] The axis scales are clearly visible. Include numbers along axes only for bottom left plot of group (a 'group' is an analysis of identical markers).
- [x] All plots are contour plots with outliers or pseudocolor plots.
- [ ] A numerical value for number of cells or percentage (with statistics) is provided.

Methodology

Sample preparation	For assessment of total FMRpolyG-GFP after ASO-CCG treatment COS7 cells were plated on 48 well plate. 2 h form plating cells were transfected with 250 ng of 100xCGG or control GFP construct at ~80% confluency with the use of Lipofectamine 3000 according to manufacturer's instructions. After 3-4 h cells were transfected with 11 nucleotide-long ASOs at 100 or 200 nM concentration with the use of Lipofectamine 3000 according to manufacturer's instructions. ASOs were denatured before transfection for 30 s in 95°C and chilled on ice. After 48 h cells were analyzed with the use of flow cytometry. Cells were trypsinized and suspended in warm PBS. Propidium iodide (PI) at final concentration 1 µg/ml was added to stain dead cells which were next excluded from analysis. GFP and PI signal in cells was analyzed with guava easyCyteTM HT System (Millipore).<br>For assessment of apoptosis induction after ASO-CCG treatment COS7 cells were plated in a 24-well plate and transfected 5 h from plating with 9 nucleotide-long ASOs and toxic-ASO at 200 nM concentration. As additional positive control cells treated with 50 µM CCCP (carbonyl cyanide 3-chlorophenylhydrazone). After 24h apoptosis of the cells was tested with Guava® MitoDamage Kit (Luminex) according to the manufacturer's protocol. Samples were analyzed on a guava easyCyte HT flow cytometer using provided MitoDamage Kit template.
Instrument	guava easyCyte™ HT Instrument
Software	guavaSoft™ software, version 3.1.1
Cell population abundance	Analyzed population of life and GFP positive cells accounted for ~15 - ~60% of all collected cells. Cells considered as positive for marker of early apoptosis (low fluorescence for MitoSense Red) accounted for ~20 - ~40% of all collected cells.
Gating strategy	For flow cytometry data analysis cells were first gated by Forward Scatter and Side Scatter (FSC/SSC). To distinguish between cells positive and negative for PI and GFP channel, control cell sample (cells non-transfected with GFP and non-stained with IP) were analyzed and utilized as background. Dead cells with strong PI signal and cells with strong expression of GFP were also analyzed as positive control. In an apoptosis assay gating strategy assigned to dedicated MitoDamage Kit template in guavaSoft™ 3.1.1 software (Luminex) was applied as presented in publicly available Guava MitoDamage Kit User Guide (Luminex, https://www.luminexcorp.com/guava-mitodamage-kit/#documentation).

- [x] Tick this box to confirm that a figure exemplifying the gating strategy is provided in the Supplementary Information.
