# Supplementary material for: Short antisense oligonucleotides alleviate the pleiotropic toxicity of RNA harboring expanded CGG repeats
Source: Nat Commun. 2021 Feb 24;12:1265. doi: 10.1038/s41467-021-21021-w (PMC7904788; doi:10.1038/s41467-021-21021-w)
Supplement: Supplementary file 2 — Supplementary Information [file 41467_2021_21021_MOESM2_ESM.pdf]

# Short antisense oligonucleotides alleviate the pleiotropic toxicity of RNA harboring expanded CGG repeats

Magdalena Derbis[1], Emre Kul[2], Daria Niewiadomska[1], Michał Sekrecki[1], Agnieszka Piasecka[1], Katarzyna Taylor[1], Renate K Hukema[3,4], Oliver Stork[2], Krzysztof Sobczak[1,*]

[1] Department of Gene Expression, Institute of Molecular Biology and Biotechnology, Faculty of Biology, Adam Mickiewicz University, Uniwersytetu Poznanskiego 6, 61-614 Poznan, Poland

[2] Department of Genetics and Molecular Neurobiology, Institute of Biology, Otto-von-Guericke University, Leipziger Str. 44, Haus 91, 39120 Magdeburg, Germany

[3] Department of Clinical Genetics, Erasmus MC, Dr. Molewaterplein 40, 3000 CA Rotterdam, The Netherlands

[4] Department of Health Care Studies, Rotterdam University of Applied Sciences, PO box 25035, 3001 HR Rotterdam, The Netherlands (current affiliation)

* To whom correspondence should be addressed. Tel.: +4861 829 5958; Fax: +4861 829 5949; Email: ksobczak@amu.edu.pl

# Table of Content


1. **Supplementary Figures**
    a. Supplementary Fig. S1. RNA-binding protein sequestration and ASO-CCG binding to rCGG$^{exp}$.
    b. Supplementary Fig. S2. The effect of ASO-CCG on translation from CGG repeat-containing transcripts.
    c. Supplementary Fig. S3. The effect of ASO-CCG on *FMR1* transcription and in vitro methods for visualization of R-loops.
    d. Supplementary Fig. S4. The effect of unassisted delivery of ASO-CCG to cells and brain of the FXTAS mouse model.
    e. Supplementary Fig. S5. Heatmap showing changes in gene expression after treatment of P90CGG mice with ASO-CCG.
    f. Supplementary Fig. S6. Off target effects of ASO-CCG and doxycycline.
    g. Supplementary Fig. S7. Representative flow cytometry gating (related to Fig. 2b and Supplementary Figs. S2a, b).
    h. Supplementary Fig. S8. Representative flow cytometry gating (related to Fig. 1d).

2. **Supplementary Tables**
    a. Supplementary Table S1. Expression of toxicity markers in brains treated with short LNA-based ASOs.
    b. Supplementary Table S2. Oligonucleotide sequences.

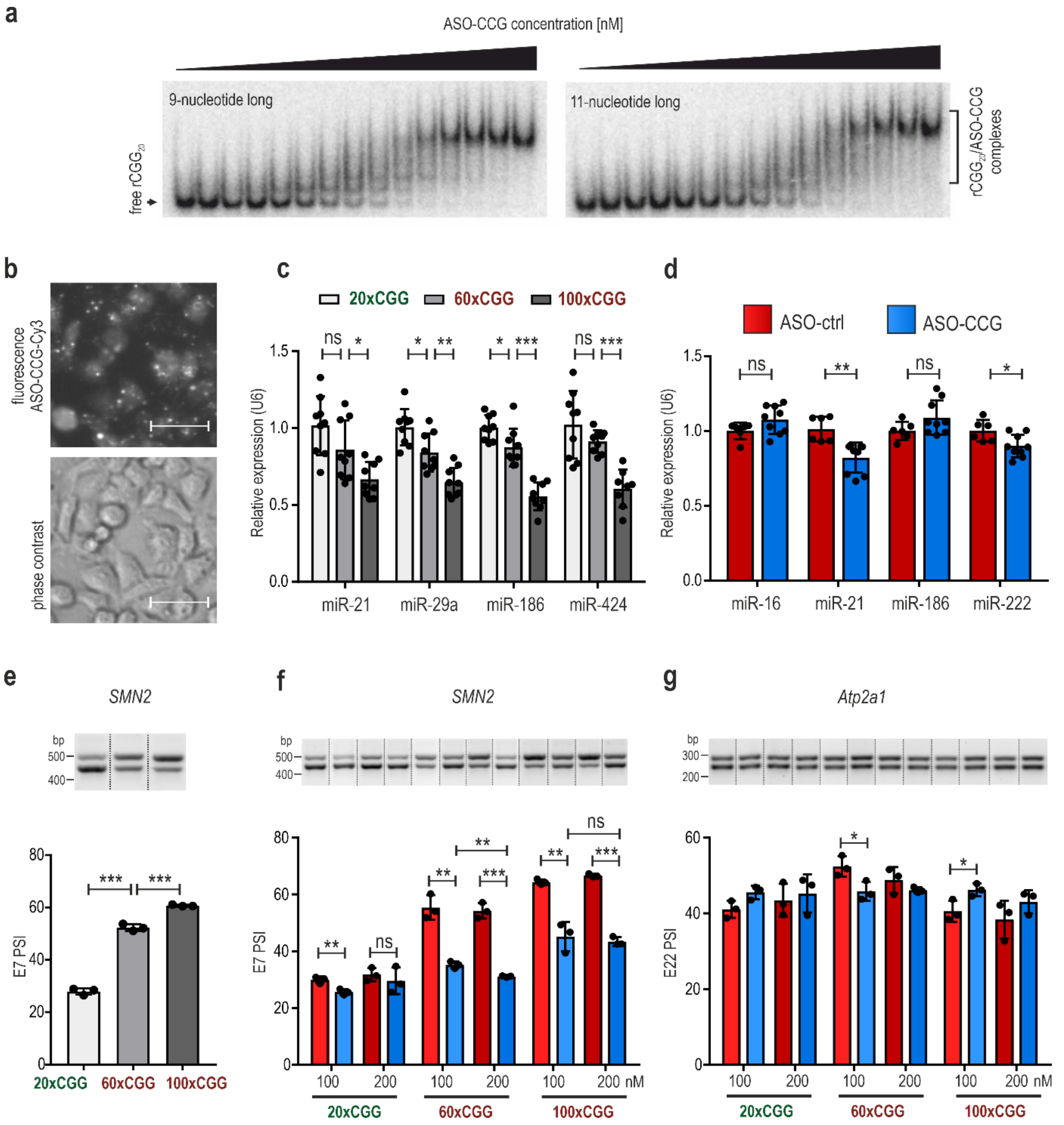


**Supplementary Fig. S1** (related to Fig. 1). **RNA-binding protein sequestration and ASO-CCG binding to rCGG$^{exp}$. a** Representative EMSA gels showing rCGG$_{20}$ and 9- and 11-nucleotide-long ASO-CCGs. Mixtures of radioactively labeled rCGG$_{20}$ and increasing amounts of ASO-CCGs with different lengths were separated on an agarose gel under native conditions. The formation of rCGG$_{20}$/ASO-CCG complexes was observed at very low oligonucleotide concentrations, especially for the shorter 9-nucleotide ASO-CCG. Experiment was repeated 3 times with similar results. **b** Representative image showing the distribution of fluorescently labeled ASO-CCG in cells. COS7 cells were transfected with 200 nM ASO-CCG-Cy3. Images were acquired 24 h post-delivery. The ASO-CCG-Cy3 signal was dispersed and present in almost every cell. Experiment was repeated two times with similar results. Scale bar, 50 µm. **c** Quantification of endogenous miRNA levels upon overexpression of rCGG$^{exp}$. COS7 cells were transfected with constructs containing ~20, ~60 or ~100 CGG repeats. Total RNA was isolated after 72 h and subjected to miRNA-specific RT-qPCR analysis. *N*=3 biologically independent

samples, each with $n$=3 technical replicates. **d** ASO-CCG effect on endogenous miRNA. COS7 cells were transfected with 200 nM ASO-CCG (9 nt). Total RNA was isolated after 48 h and subjected to miRNA-specific RT-qPCR analysis. $N$=3 biologically independent samples, each with $n$=3 technical replicates. Note that without rCGG$^{exp}$ overexpression, the miRNA level was slightly decreased or unchanged after ASO-CCG delivery. **e** Analysis of SAM68-dependent alternative splicing events in the *SMN2* minigene upon overexpression of rCGG$^{exp}$. COS7 cells were cotransfected with constructs containing ~20, ~60 or ~100 CGG repeats and the *SMN2* minigene. Total RNA was isolated after 48 h and subjected to RT-PCR analysis ($N$=3 biologically independent samples). **f** Analysis of SAM68-dependent alternative splicing events in *SMN2* minigenes upon delivery of different concentrations of ASO-CCG and overexpression of normal or expanded CGG repeats. COS7 cells were cotransfected with constructs containing ~20, ~60 or ~100 CGG repeats and the *SMN2* minigene. A 48 h incubation period with ASO-CCG (100 or 200 nM, 11 nt) was followed by RT-PCR analysis. $N$=3 biologically independent samples. **g** Analysis of SAM68-independent alternative splicing events in the *Atp2a1* minigene upon delivery of different concentrations of ASO-CCG and overexpression of normal or expanded CGG repeats. COS7 cells were cotransfected with constructs containing ~20, ~60 or ~100 CGG repeats and the *Atp2a1* minigene. A 48 h incubation period with ASO-CCG (100 or 200 nM, 11 nt) was followed by RT-PCR analysis. $N$=3 biologically independent samples. **c**, **e** light grey (silver) bars, cells overexpressing ~20 CGG repeats; grey (ash) bars, ~60 CGG repeats; dark grey (anthracite) bars, ~100 CGG repeats. **d**, **f**, **g** red bars, ASO-ctrl; blue bars, ASO-CCG, light colors, 100 nM; dark colors, 200 nM. **c**, **d** The values shown in graphs are the means of indicated $N$ and $n$ with the SDs. **e**-**g** The values shown in the graphs are the mean percentages of indicated $N$ for the exon 7 (**e**, **f**) or exon 22 (**g**) inclusion isoform, with the SDs. Samples were derived from the same experiment and processed in parallel on different gels. PSI, the percent of spliced in. Presented gels were cropped. **c**-**g** Green, cells overexpressing normal CGG repeats (20xCGG); red, expanded CGG repeats (60- and 100xCGG). Statistical analysis of the data in graphs was based on two-tailed unpaired Student's *t*-test; *, $p$<0.05; **, $p$<0.01; ***, $p$<0.001; ns, non-significant. Source data are provided as a Source Data file.

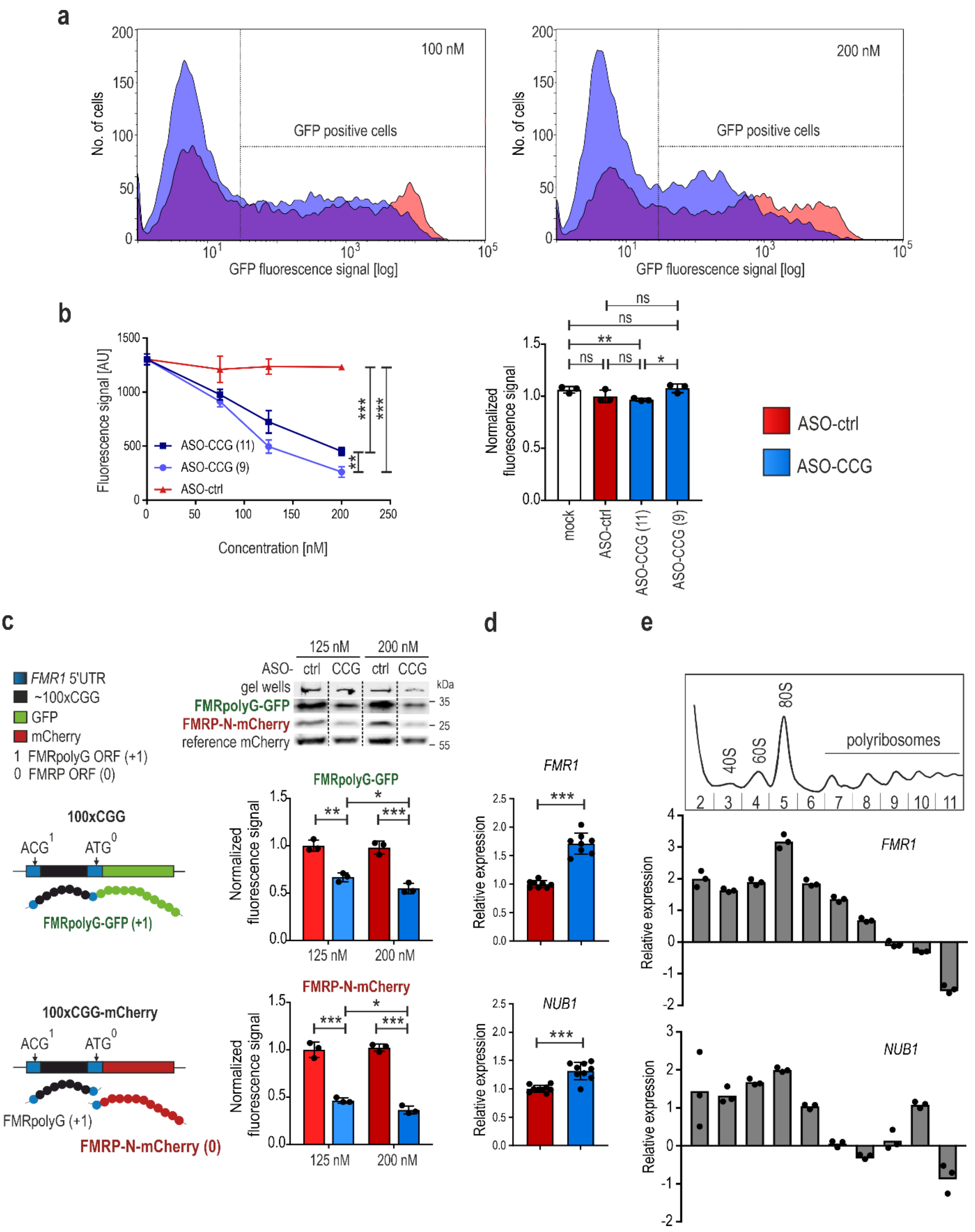


**Supplementary Fig. S2** (related to Fig. 2). **The effect of ASO-CCG on translation from CGG repeat-containing transcripts. a** Representative histograms of the FMRpolyG-GFP signal distribution in the flow cytometric assay. COS7 cells transfected with the 100xCGG construct and ASO-CCG or ASO-ctrl (100 and 200 nM, 11 nt) were analyzed after with flow cytometry. The fluorescence signal in GFP-positive cells was measured with dead cells stained with propidium iodide excluded. Note that treatment with ASO-CCG (blue) decreased the number of cells exhibiting a high GFP signal and increased the number of GFP-negative cells. **b** Comparison of the effects of 9- and 11-nucleotide

ASO-CCG on FMRpolyG-GFP biosynthesis. COS7 cells transfected with the 100xCGG construct and 9- (light blue/dots) or 11- (dark blue/squares) nucleotide long ASO-CCG and 11-nucleotide long ASO-ctrl (red/triangles; all ASOs, 75, 125 and 200 nM) were analyzed with flow cytometry after 48 h. The fluorescence signal of GFP-positive cells was measured with dead cells stained with propidium iodide excluded (*N*=3 biologically independent samples). Quantification of the observed signals revealed that 9- and 11-nucleotide ASO-CCG (200 nM) exerted similar effects in comparison to those of ASO-ctrl; however, 9-nucleotide ASO-CCG had a significantly greater effect than 11-nucleotide ASO-CCG. Signals of COS7 cells transfected with control GFP construct and ASO-CCG (200 nM) were also measured (*N*=3 biologically independent samples). White bar, mock (transfection agent). **c** Quantification of the levels of soluble FMRpolyG-GFP and FMRP equivalent (FMRP-N-mCherry) upon ASO-CCG delivery. COS7 cells were cotransfected with the 100xCGG, 100xCGG-mCherry and reference mCherry constructs and either ASO-CCG or ASO-ctrl (125 and 200 nM, 11 nt). Cells were lysed after 48 h, and proteins were separated with SDS-PAGE. A scanner was used to detect fluorescence signals directly in gels. Left, schematics of the 100xCGG and 100xCGG-mCherry constructs, including their protein products. Constructs contain the 5'UTR of the *FMR1* gene (blue bars) with ~100 CGG repeats (black bar) fused with the GFP (green bar) or mCherry (red bar) coding sequence. FMRP equivalent, FMRP-N-mCherry, protein which translation starts from FMRP-specific start codon in 100xCGG-mCherry genetic construct. Right, quantification of signals detected in gels for FMRpolyG-GFP and FMRP equivalent. *N*=3 biologically independent samples. ASO-ctrl and ASO-CCG samples were derived from the same experiment and processed in parallel on different gels. Presented gels were cropped. **d** Quantification of the total level of mRNAs containing short CGG repeats. COS7 cells were transfected with 200 nM ASO-CCG (9 nt). RT-qPCR analysis of mRNA levels was performed after 48 h with primers specific for COS7-endogenous *FMR1* and *NUB1* transcripts containing 12 CGG repeats (*N*=3 biologically independent samples, each with *n*=3 technical replicates). The values shown in the graph are the means of *N* and *n*, with the SDs. **e** Association of mRNAs containing short CGG repeats with ribosomes. COS7 cells were transfected with 200 nM ASO-CCG (9 nt). After 48 h, cell extracts were fractionated on a linear sucrose gradient (15-45%). Total RNA was isolated from the collected fractions containing free mRNAs, monoribosomes and polyribosomes and was then analyzed using RT-qPCR. Upper, graph showing the RNA abundance in the fractions, as measured at 254 nm. Lower, RT-qPCR analysis of the endogenous *FMR1* and *NUB1* mRNA levels in ASO-CCG-treated COS7 cells, where the reference sample is the corresponding fraction in ASO-ctrl-treated cells and the reference gene is *GAPDH*. Graphs present means of *n*=3 technical replicates. Similar results were obtained in 2 independent experiments. **b**-**d** Red bars, ASO-ctrl; blue bars, ASO-CCG; light colors, 100 nM; dark colors, 200 nM. **b**, **c** The values shown in the graphs are the means of *N* independent experiments, with the SDs. **b**, **c**, **d** Statistical analysis was based on two-tailed unpaired Student's *t*-test; *, p<0.05; **, p<0.01; ***, p<0.001; ns, non-significant. **b**-**e** Source data are provided as a Source Data file.

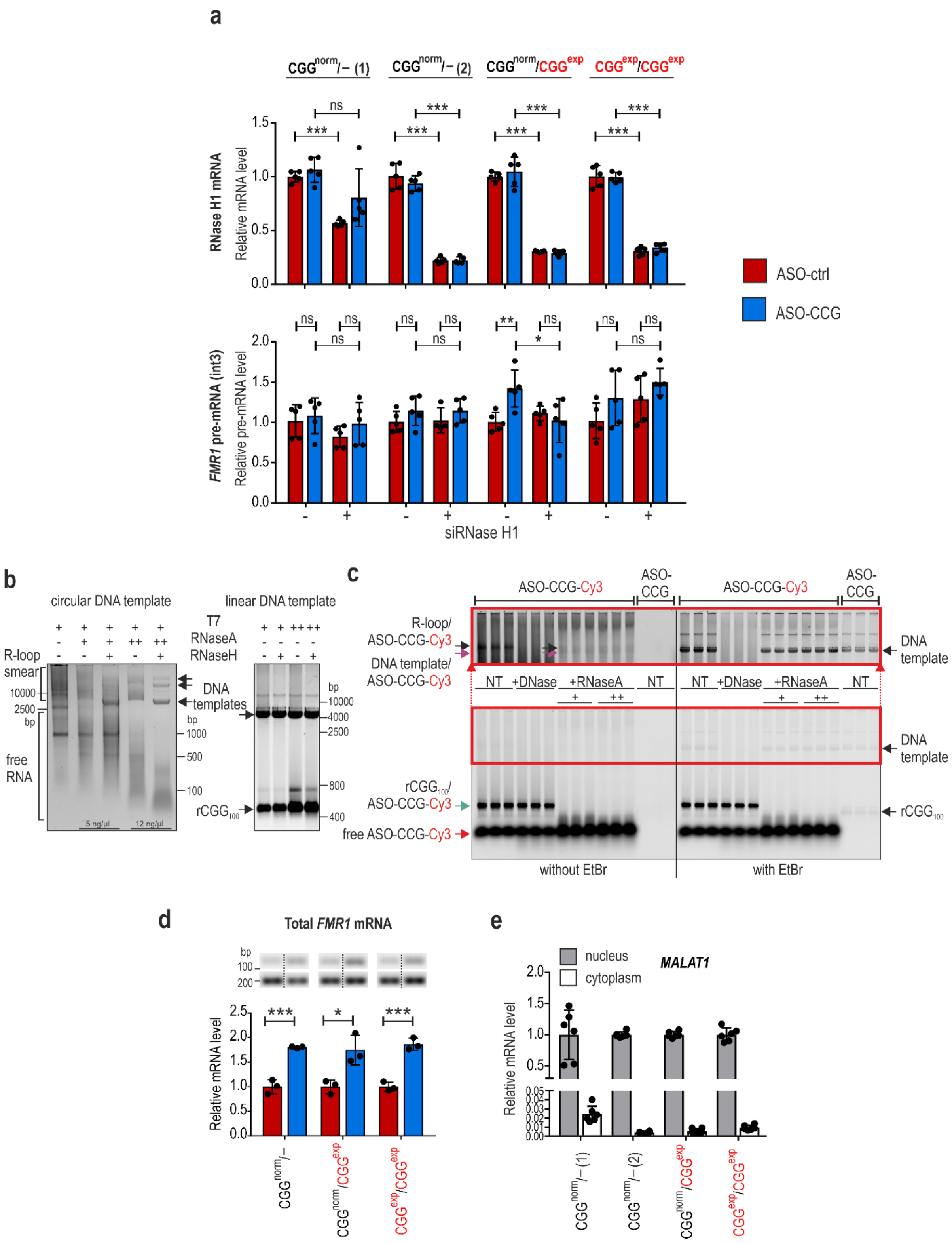


**Supplementary Fig. S3** (related to Fig. 3). **The effect of ASO-CCG on *FMR1* transcription and in vitro methods for visualization of R-loops. a** Analysis of *FMR1* pre-mRNA and RNase H1 mRNA levels in ASO-CCG-treated fibroblasts upon RNase H1 insufficiency. Fibroblasts were transfected with siRNA against RNase H1 and, after 24 h, with 200 nM ASO-CCG (9 or 11 nt). After an additional 48 h, RT-qPCR analysis was performed with primers specific for the RNase H1 mRNA showing the downregulation level (upper) and for *FMR1* intron 3 sequence showing the pre-mRNA level (lower). *N*=5 biologically independent samples. **b** Approaches for monitoring the effect of R-loops

on transcription. Visualization of R-loops in 5'-end of *FMR1* was performed using two approaches. Left, In vitro transcription performed using the non-digested 100xCGG genetic construct (circular plasmid). The reactions were performed at 37°C for 2 h and then were treated with 1 µl of RNase A („+'', 5 ng/µl; „++'', 12 ng/µl) and 1 U of RNase H (+) or equal amount of 50% glycerol in untreated samples (-) for an additional 30 min at 37°C. Reactions were stopped and nucleic acids were extracted with phenol-chloroform and precipitated with 96% ice cold ethanol and 3 mM sodium acetate. Pelleted nucleic acids were resuspended in the water with 6 x BLUE DNA Loading buffer (Blirt) and analyzed on 1% agarose gels with ethidium bromide (0.5 µg/ml) run in 1 x Tris-Borate-EDTA buffer at 70 V for 2 h. To visualize the nucleic acid products the agarose gels were scanned using Amersham Typhoon RGB Biomolecular Imager. Nucleic acids with intercalated ethidium bromide were detected using Cy3 filter. Right, In vitro transcription performed on the 100xCGG genetic construct digested with Avr II recognizing restriction site downstream putative R-loop forming sequence and $CGG^{exp}$ (allow for transcription of whole exon 1 of *FMR1*). The reactions were performed using different amount of T7 RNA polymerase ("+" or "++") at 37°C for 20 min in the presence of 1 U of RNase H (+) or 50% glycerol in the untreated samples (-). Reactions were stopped by the addition of 6 x BLUE DNA loading buffer containing 20 mM EDTA (Blirt) and analyzed on 1% agarose gel as described above. Experiment was repeated 3 times with similar results. **c** In vitro transcription experiment showing interaction of ASO-CCG with R-loops and a sense strand of DNA template with $CGG_{100}$. In vitro transcription was performed on the ~500 ng of 100xCGG construct digested with Avr II at 37°C for 20 min in the presence of ASO-CCG or fluorescently labelled ASO-CCG-Cy3 (indicated above the gel). Then samples were digested with either 2 U of DNase TURBO (DNase) or 1 µl of RNase A („+'', 0.3 µg/µl; „++'', 5 µg/µl) for 30 min on ice. Control samples were treated with the 50% glycerol (NT). Reactions were stopped by addition of 6x BLUE DNA Loading buffer (Blirt) and analyzed on 1% agarose gels run in 1 x Tris-Borate-EDTA buffer at 70 V for 3 h. To visualize $CGG_{100}$-containing nucleic acids the agarose gels were scanned using Amersham Typhoon RGB Biomolecular Imager and fluorescent signal coming from ASO-CCG-Cy3 was detected using Cy3 filter (without EtBr). Then gels were stained with ethidium bromide (0.5 µg/ml) for 20 min and scanned again to visualize DNA templates and all RNA products (with EtBr). Area of gel marked in red is presented above with higher exposition. Experiment was repeated 3 times with similar results. **d** Analysis of the total *FMR1* mRNA level in ASO-CCG-treated fibroblasts. Fibroblasts were transfected with 200 nM ASO-CCG (11 nt), and RT-PCR analysis was performed after 48 h with primers specific for the *FMR1* exonic sequence ($N$=3 biologically independent samples). The values shown in the graph are the means of $N$ independent experiments, with the SDs. **e** Quantification of nucleus-specific transcripts in the nuclear and cytoplasmic fractions. Fibroblasts were transfected with 200 nM ASO-CCG (9 nt). After 48 h, nucleocytoplasmic fractionation was performed, followed by RT-qPCR analysis with primers specific for the nucleus-specific *MALAT1* transcript. Grey bars, nucleus; white bars, cytoplasm. Graph presents mean of $N$=3 biologically independent samples, each with $n$=2 technical replicates, with the SDs. **a**, **d**, **e** The cell lines used contained an allele with a normal CGG repeat length (lines (1) and (2)), two alleles–one with a normal CGG repeat length and one with $CGG^{exp}$ and two alleles with $CGG^{exp}$. Statistical analysis was based on two-tailed unpaired Student's *t*-test; *, $p<0.05$; **, $p<0.01$; ***, $p<0.001$; ns, non-significant. **a**, **d** Red bars, ASO-ctrl; blue bars, ASO-CCG. The values shown in the graphs are the means of $N$, with the SDs. Presented gels were cropped. **b**, **c** Experiments were repeated 3 times with similar results. **a**-**e** Source data are provided as a Source Data file.

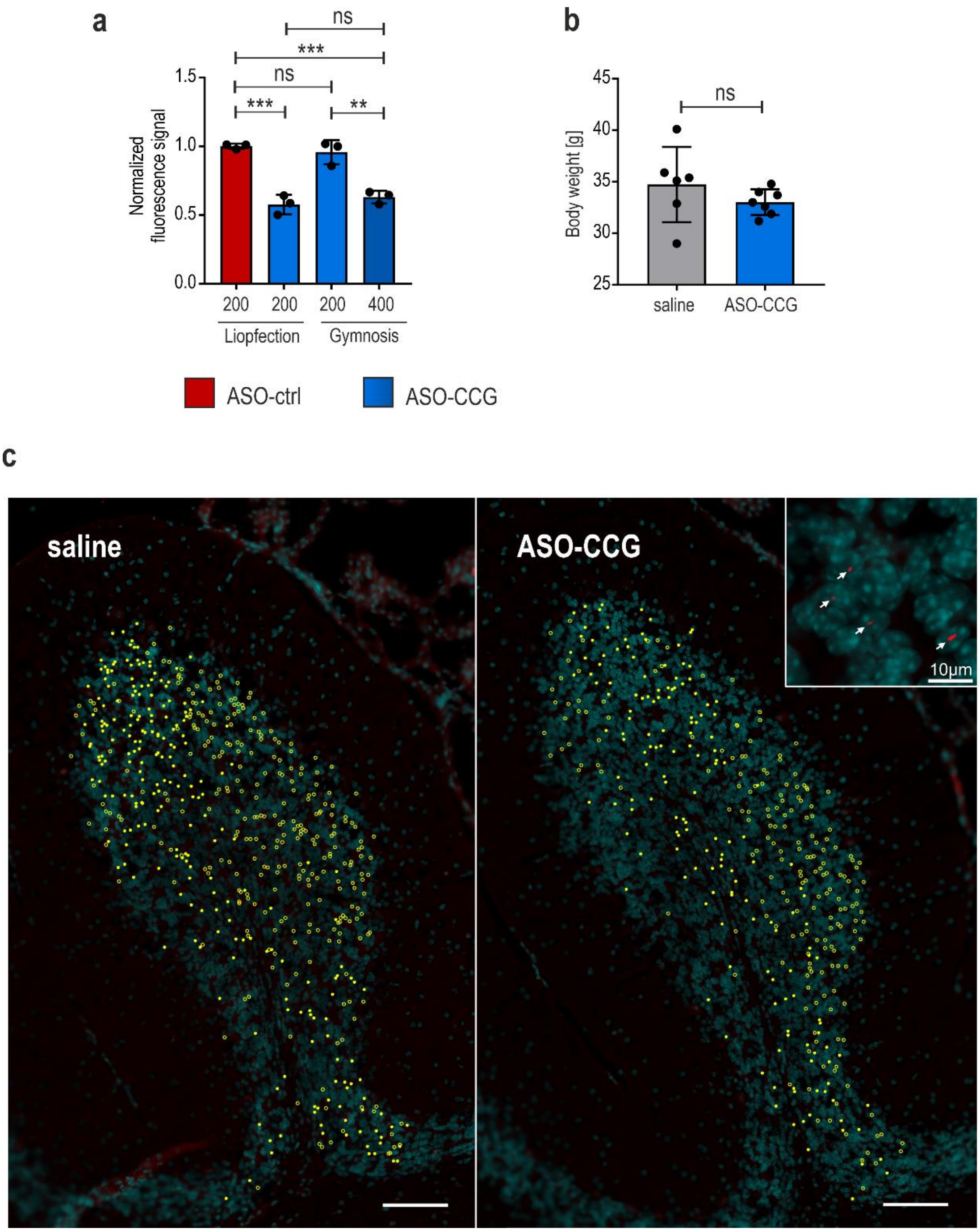


**Supplementary Fig. S4** (related to Fig. 4). **The effect of unassisted delivery of ASO-CCG to cells and brain of the FXTAS mouse model. a** Comparison of the effect of ASO-CCG delivered via lipofection or gymnosis on FMRpolyG-GFP biosynthesis. COS7 cells were transfected with 100xCGG construct. ASO-CCG (9 nt) was delivered to cells via lipofection at a concentration of 200 nM (light blue bar) or directly in the culture medium (gymnosis) at a concentration of 200 (light blue bar) or 400 nM (dark blue bar). Cells were analyzed with flow cytometry after 48 h. The fluorescence signal from GFP-positive cells was measured with dead cells stained with propidium iodide excluded. The observed signals were quantified. Red bar, ASO-ctrl, 200 nM. The values shown in the graph are the means of *N*=3 biologically independent samples, with the SDs. **b** Body weight of P90CGG mice subjected to behavioral tests. As body weight differences may potentially affect the performance on rotarod tests, the body weights of all mice were measured at the end of the behavioral testing

by placing the mice on a laboratory scale. No differences were observed between ASO-CCG (11 nt)-treated and saline-treated mice (p=0.1991). Grey bar, saline-treated animals, *N*=6; blue bar, ASO-CCG treated animals, *N*=7. The values shown on the bars are the means *N* animals per group. The error bars indicate SDs. **c** Representative image of FMRpolyG staining. The granule cell layer of cerebellar lobule X from P90CGG mice was stained using the 8FM antibody specific for FMRpolyG. The yellow marks indicate FMRpolyG foci. Scale bar, 100 µm; magnified part, scale bar, 10 µm. Presented images were pseudo-colored and merged. Micrograph is an extension of Fig. 4f and subject to the *N* indicated for that figure. **a**, **b** Statistical analysis was based on Student's *t*-test; *, $p<0.05$; **, $p<0.01$; ***, $p<0.001$; ns, non-significant. Source data are provided as a Source Data file.

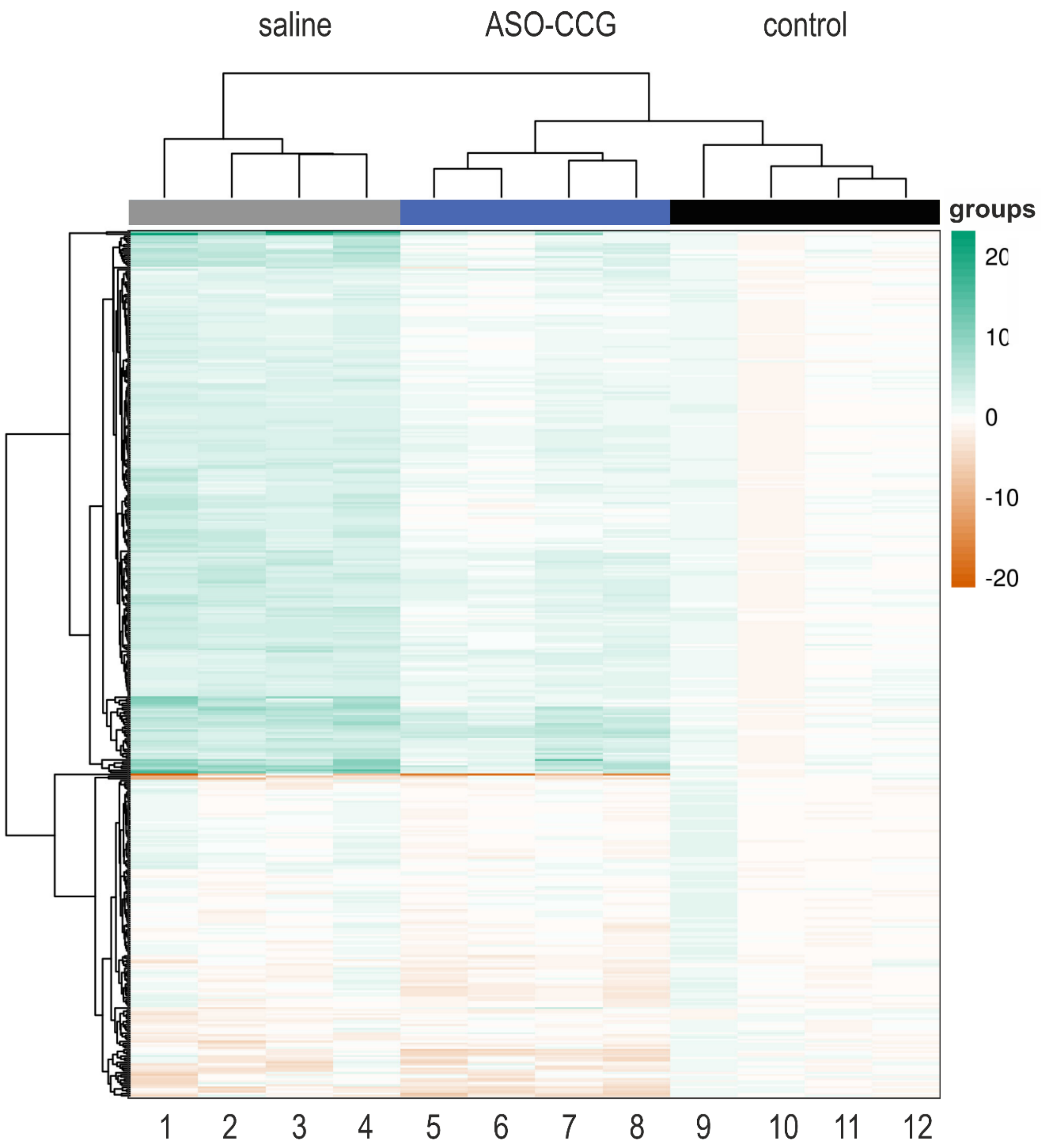


**Supplementary Fig. S5** (related to Fig. 5). **Heatmap showing changes in gene expression in striatum after treatment of P90CGG mice with ASO-CCG.** Heatmap was created based on differential expression analysis results which was performed with the use of voom+limma pipeline as described in Material and Methods section (*p*-value was generated using moderated *t*-statistic and adjusted for multiple testing using Benjamini-Hochberg's method, adj.P.Val). Genes significantly changed (adj.P.Val <0.05) in saline-treated P90CGG mice compared to control mice were included in the analysis. For every gene (row) and every sample containing a given gene (column), a Z-score was calculated using the mean and standard deviation of four control samples. Set of genes present in twelve samples were visualized using three color heatmap. Script written in R language which was used to generate heatmap is provided as a separate Supplementary file (Supplementary R script to generate heatmap).

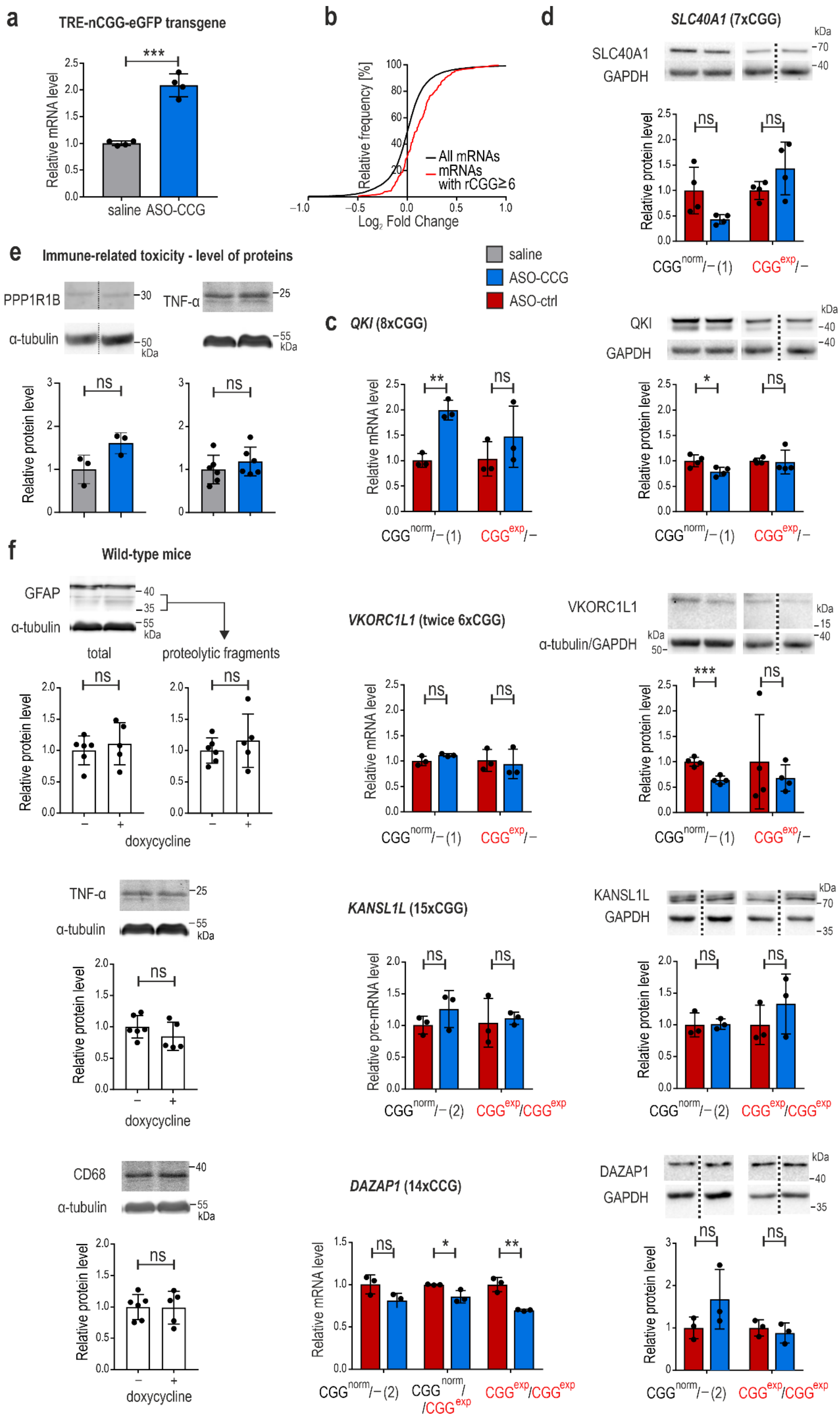
a
TRE-nCGG-eGFP transgene
***
Relative mRNA level
saline ASO-CCG
b
Relative frequency [%]
All mRNAs
mRNAs with rCGG≥6
Log2 Fold Change
d
SLC40A1 (7xCGG)
SLC40A1
GAPDH
kDa
Relative protein level
ns
CGGnorm/− (1)
CGGexp/−
e
Immune-related toxicity - level of proteins
PPP1R1B
TNF-α
α-tubulin
saline
ASO-CCG
ASO-ctrl
c
QKI (8xCGG)
QKI
GAPDH
**
*
Relative mRNA level
f
Wild-type mice
GFAP
α-tubulin
total
proteolytic fragments
doxycycline
VKORC1L1 (twice 6xCGG)
VKORC1L1
α-tubulin/GAPDH
***
KANSL1L (15xCGG)
KANSL1L
Relative pre-mRNA level
CGGnorm/− (2)
CGGexp/CGGexp
CD68
DAZAP1 (14xCCG)
DAZAP1
CGGnorm/CGGexp

**Supplementary Fig. S6** (related to Fig. 5). **Off target effects of ASO-CCG and doxycycline.** **a** Quantification of the mRNA level of the TRE-nCGG-eGFP transgene in mice treated with ASO-CCG. RNA was isolated from striatal tissue of P90CGG mice treated with saline or ASO-CCG (11 nt). RT-qPCR analysis was performed with primers specific for the transgene mRNA. *N*=4. **b** Analysis of expression changes in CGG repeat-containing genes in ASO-CCG-treated P90CGG mice. The results of RNA-seq analysis of RNA isolated from the striatum of saline-treated and ASO-CCG-treated mice (*N*=4 for each group). Global comparison of gene expression between these two groups of mice showed a significant increase in the expression of 31 genes containing at least 6 CGG repeats (red line) compared to all analyzed genes (black line; p<0.001, two-tailed Mann-Whitney test). **c** Analysis of the expression of genes containing short CGG repeats in 5'UTRs in ASO-CCG-treated fibroblasts. Fibroblasts were transfected with 200 nM ASO-CCG (9 or 11 nt), and RT-qPCR analysis was performed after 48 h with primers specific for mRNA or pre-mRNA sequences of selected genes. *N*=3 biologically independent samples. **d** Off-target effect of ASO-CCG on protein level in fibroblasts. Fibroblasts were transfected with 200 nM ASO-CCG (9 or 11 nt), and western blot analysis of proteins encoded by genes containing short CGG or CCG repeats in 5'UTRs was performed after 48 h with specific antibodies. *N*=4 biologically independent samples for SLC40A1, QKI, VKORC1L1 and *N*=3 for KANSL1L and DAZAP1. **e** Steady state level of immune system-related toxicity markers in ASO-CCG-treated P90CGG mice (western blot). Cortex: PPP1RB1, *N*=3 animals. Hippocampus: TNF-α, *N*=6 animals. Samples were derived from the same experiment and processed in parallel on different gels. **f** The effect of doxycycline treatment on immune system-related markers in wild-type mice brains. Western blot analysis was performed on hippocampus tissue lysates derived from C57BL/6JBomTac mice treated or not treated with doxycycline for 12 weeks (*N*=5 and 6, respectively). Samples were derived from the same experiment and processed in parallel on different gels. **a**, **c**-**e** Grey bars, saline-treated animals; red bars, ASO-ctrl-treated cells; blue bars, ASO-CCG-treated animals/cells. **d-f** All presented blots were cropped. **a**, **c**-**f** Graphs present means of indicated *N*, with the SDs. Statistical analysis was based on two-tailed unpaired Student's *t*-test; *, *p*<0.05; **, *p*<0.01; ***, *p*<0.001; ns, non-significant. **a**-**f** Source data are provided as a Source Data file.

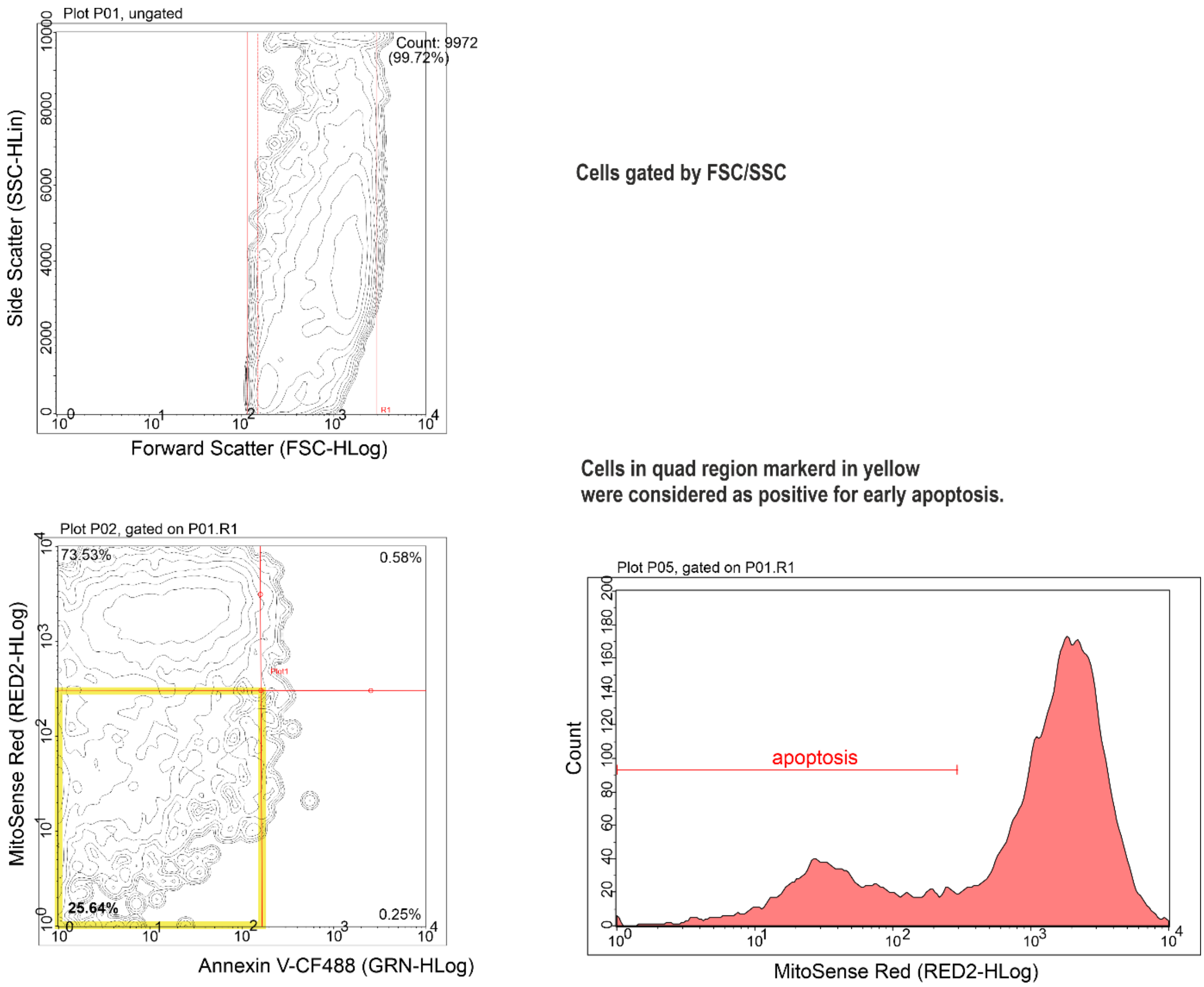


**Supplementary Fig. S7** (related to Fig. 1d) **Representative flow cytometry gating.** Cells were stained with the use of Guava® MitoDamage Kit (Luminex) and analyzed with the use of parameters and gating strategy assigned to dedicated MitoDamage Kit template from guavaSoft™ software, version 3.1.1 (Luminex). Gating strategy is presented in publicly available Guava MitoDamage Kit User Guide (Luminex, https://www.luminexcorp.com/guava-mitodamage-kit/#documentation). Cells were first gated by Forward Scatter and Side Scatter (FSC/SSC). Next cells were plotted considering MitoSense Red fluorescence and Annexin V fluorescence. Cells with lower fluorescence for MitoSense Red were considered as positive for early apoptosis. MitoSense Red fluorescence is also presented on the histogram.

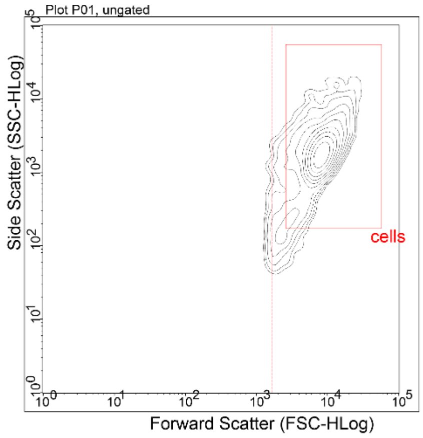


**Cells gated by FCS/SSC**

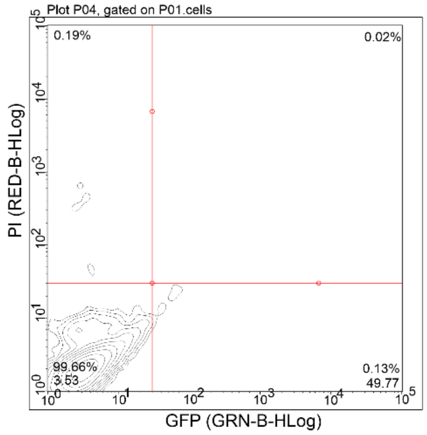


**Cells non-stained with PI and non-transfected with GFP**

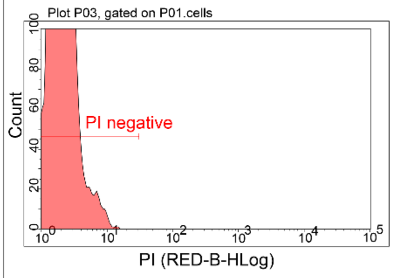


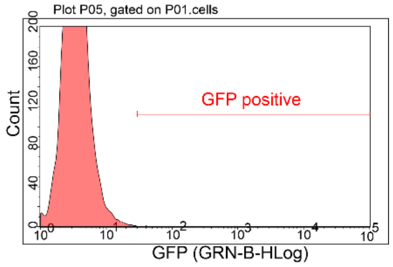


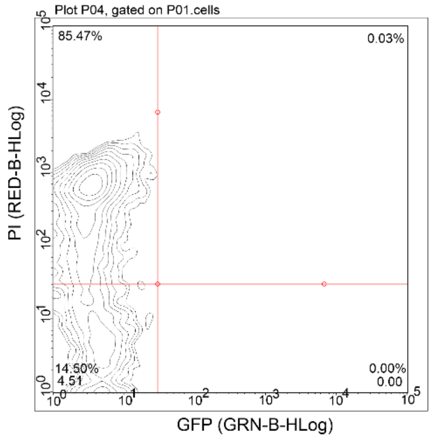


**Dead cells stained with PI and non-transfected with GFP**

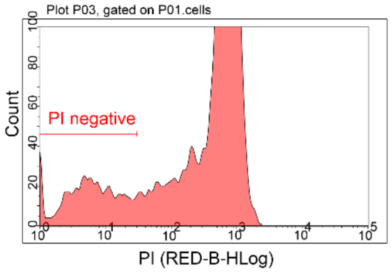


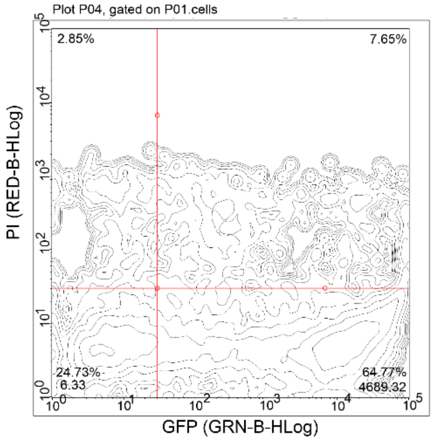


**Cells with strong expression of GFP and stained with PI**

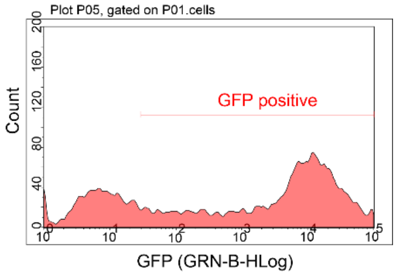

**Supplementary Fig. S8** (related to Fig. 2b and Supplementary Figs. S2a, b) **Representative flow cytometry gating.** For flow cytometry data analysis cells were first gated by Forward Scatter and Side Scatter (FSC/SSC). To distinguish between cells positive and negative for PI and GFP channel, control cell sample (cells non-transfected with GFP and non-stained with PI) were analyzed and utilized as background. Dead cells with strong PI signal and cells with strong expression of GFP were also analyzed as positive control.

**Supplementary Table S1. Expression of toxicity markers in brains treated with short LNA-based ASOs.**

| Gene ID | Gene name | Protein name | Expression in cortex (logFC) | Expression in striatum (logFC) | Notes | Refs |
|---|---|---|---|---|---|---|
| **Immune system-related markers** | | | | | | |
| ENSMUSG00000031639 | *Tlr3* | Toll-like receptor 3 | 0.09875 | -0.08376 | Receptor for viral and synthetic dsRNAs (double-stranded RNAs); induce IFN-α and 2′-5′-oligoadenylate synthase (OAS); triggered by m5C, m5U, s2U, m6A | [1,2] |
| ENSMUSG00000044583 | *Tlr7* | Toll-like receptor 7 | 0.36950 | **0.89715** | Receptor for viral ssRNAs (single-stranded RNAs) and nucleosides; triggered by 2′-O-methyl, thymidine, 2′-F, m5C, m5U, s2U, m6A | [3–8] |
| ENSMUSG00000027776 | *Il12a* | Interleukin-12 subunit alpha | 0.31054 | N/A | | [9] |
| ENSMUSG00000037523 | *Mavs* | Mitochondrial antiviral-signaling protein | 0.07849 | 0.03898 | | [10] |
| ENSMUSG00000024079 | *Eif2ak2* | Interferon-induced, double-stranded RNA-activated protein kinase | 0.18167 | 0.31245 | | [11] |
| ENSMUSG00000001166 | *Oas1c* | Inactive 2'-5' oligoadenylate synthetase 1C | 0.10811 | N/A | IFN-stimulated gene (ISGs) | [12] |
| ENSMUSG00000023341 | *Mx2* | Interferon-induced GTP-binding protein Mx2 | N/A | 0.15767 | IFN-stimulated gene (ISGs) | [13] |
| ENSMUSG00000074896 | *Ifit3* | Interferon-induced protein with tetratricopeptide repeats 3 | -0.14037 | -0.17343 | Gene expression altered in mice treated intracerebroventricularly with phosphorothioate, 2'-O-methyl modified ASOs | [14] |
| ENSMUSG00000062488 | *Ifit3b* | Interferon-induced protein with tetratricopeptide repeats 3B | 0.02668 | -0.10424 | Gene expression altered in mice treated intracerebroventricularly with phosphorothioate, 2'-O-methyl modified ASOs | [14] |
| ENSMUSG00000029561 | *Oasl2* | 2'-5'-oligoadenylate synthase-like protein 2 | -0.28093 | -0.14089 | Gene expression altered in mice treated intracerebroventricularly with phosphorothioate, 2'-O-methyl modified ASOs | [14] |
| ENSMUSG00000046718 | *Bst2* | Bone marrow stromal antigen 2 | 0.31572 | 0.46018 | Gene expression altered in mice treated intracerebroventricularly with phosphorothioate, 2'-O-methyl modified ASOs | [14] |

| | | | | | | |
|---|---|---|---|---|---|---|
| ENSMUSG00000026728 | *Vim* | Vimentin | **0.44498** | **0.81135** | Astrocyte marker | [15] |
| ENSMUSG00000024397 | *Aif1* | Allograft inflammatory factor 1 | -0.14592 | 0.70354 | Microglia Marker | [16] |
| ENSMUSG00000030786 | *Itgam* | Integrin alpha-M | -0.04963 | -0.00805 | Microglia Marker | [16] |
| ENSMUSG00000026395 | *Ptprc* | Receptor-type tyrosine-protein phosphatase C | 0.13587 | **1.04583** | Microglia Marker | [16] |
| ENSMUSG00000054675 | *Tmem119* | Transmembrane protein 119 | -0.17370 | **-0.36652** | Microglia Marker | [16] |
| ENSMUSG00000046805 | *Mpeg1* | Macrophage-expressed gene 1 protein | **0.60365** | **1.46506** | Macrophage-specific marker, neuronal inflammation | [17] |
| ENSMUSG00000018774 | *Cd68* | CD68 | **0.71095** | **0.92049** | Microglia Marker | |
| ENSMUSG00000020932 | *Gfap* | Glial fibrillary acidic protein | **0.88630** | **2.81900** | Neuronal inflammation | |
| ENSMUSG00000040296 | *Ddx58* | Antiviral innate immune response receptor RIG-I | 0.01676 | 0.07127 | Target: 5′-tri- and -diphosphate, AU-rich, dsRNA; triggered by 2′-O-methyl, 2′-F, m7G cap, m5U, s2U; 5′PO | [18,19] |
| ENSMUSG00000026896 | *Ifih1* | Interferon-induced helicase C domain-containing protein 1 | 0.18415 | 0.11050 | Target: long dsRNA; To avoid: use sequences<300 bp | [19] |
| ENSMUSG00000037860 | *Aim2* | Interferon-inducible protein AIM2 | -0.13132 | 0.10330 | Target: dsDNA; To avoid: use sequences<40 bp | [19] |
| ENSMUSG00000032344 | *Cgas* | Cyclic GMP-AMP synthase | N/A | 0.58604 | Target: G-flanked, short dsDNAG-rich, Y-shaped DNA; triggered by ssDNA sequences that can form dsDNA | [19,20] |
| **Apoptosis markers** | | | | | | |
| ENSMUSG00000057329 | *Bcl2* | Apoptosis regulator Bcl-2 | 0.10788 | -0.03042 | Marker of apoptosis | [21] |
| ENSMUSG00000003873 | *Bax* | Apoptosis regulator BAX | -0.05501 | 0.34575 | Marker of apoptosis | [21] |
| ENSMUSG00000031628 | *Casp3* | Caspase-3 | -0.00978 | 0.02458 | Marker of apoptosis if active (cleaved) | [22] |
| ENSMUSG00000026496 | *Parp1* | Poly(ADP-ribose) polymerase 1 | -0.00308 | 0.01520 | Marker of apoptosis if active, DNA damage | |
| ENSMUSG00000059552 | *Trp53* | Cellular tumor antigen p53 | 0.01905 | -0.06881 | Marker of apoptosis | [23] |
| **Others** | | | | | | |
| ENSMUSG00000026163 | *Sphkap* | A-kinase anchor protein SPHKAP | -0.01045 | -0.03413 | Gene expression altered in mice treated intracerebroventricularly with phosphorothioate, 2'-O-methyl modified ASOs | [14] |

| | | | | | | |
|---|---|---|---|---|---|---|
| ENSMUSG00000033880 | *Lgals3bp* | Galectin-3-binding protein | 0.18769 | 0.66143 | Gene expression altered in mice treated intracerebroventricularly with phosphorothioate, 2'-O-methyl modified ASOs | 14 |
| ENSMUSG00000040164 | *Kcns1* | Potassium voltage-gated channel subfamily S member 1 | -0.08578 | -0.10361 | Gene expression altered in mice treated intracerebroventricularly with phosphorothioate, 2'-O-methyl modified ASOs | 14 |
| ENSMUSG00000030747 | *Dgat2* | Diacylglycerol O-acyltransferase 2 | -0.04788 | 0.04589 | Gene expression altered in mice treated intracerebroventricularly with phosphorothioate, 2'-O-methyl modified ASOs | 14 |
| ENSMUSG00000034327 | *Kctd9* | BTB/POZ domain-containing protein KCTD9 | 0.21576 | 0.07963 | Gene expression altered in mice treated intracerebroventricularly with phosphorothioate, 2'-O-methyl modified ASOs | 14 |
| ENSMUSG00000032528 | *Vipr1* | Vasoactive intestinal polypeptide receptor 1 | 0.05154 | 0.03767 | Gene expression altered in mice treated intracerebroventricularly with phosphorothioate, 2'-O-methyl modified ASOs | 14 |
| ENSMUSG00000035929 | *H2-Q4* | Histocompatibility 2, Q region locus 4 | 0.25618 | 0.65936 | Gene expression altered in mice treated intracerebroventricularly with phosphorothioate, 2'-O-methyl modified ASOs | 14 |
| ENSMUSG00000061718 | *Ppp1r1b* | Protein phosphatase 1 regulatory subunit 1B | 0.47384 | -0.14510 | Marker of spiny neurons | |
| ENSMUSG00000004891 | *Nes* | Nestin | -0.01026 | 0.15477 | Marker of neural stem/progenitor cell | |

The Table contains information about markers of: immune-related toxicity, apoptosis and other markers induced by short nucleic acids and their analogs according to literature data. Results of differential gene expression analysis of RNA-seq data from striatal and cortical tissue of P90CGG mouse for ASO-CCG-treated vs saline-treated group for those markers were enclosed. Analysis was performed with the use of voom+limma pipeline as described in Material and Methods section. *p*-value was generated using moderated *t*-statistic and adjusted for multiple testing using Benjamini-Hochberg's method (adj.P.Val). Presented values are bolded if statistically significant (adj.P.Val <0.05). For markers identified in human cells according to cited literature, murine orthologs (Ensembl database) were applied. GeneID, gene identification number from Ensembl database; logFC, log2 fold change. 1. Alexopoulou, L., Holt, A. C., Medzhitov, R. & Flavell, R. A. Recognition of double-stranded RNA and activation of NF-κB by Toll-like receptor 3. *Nature* **413**, 732–738 (2001). 2. Karikó, K., Bhuyan, P., Capodici, J. & Weissman, D. Small Interfering RNAs Mediate Sequence-Independent Gene Suppression and Induce Immune Activation by Signaling through Toll-Like Receptor 3. *J. Immunol.* **172**, 6545–6549 (2004). 3. Agrawal, S. & Kandimalla, E. R. Synthetic agonists of Toll-like receptors 7, 8 and 9. in *Biochemical Society*

*Transactions* **35**, 1461–1467 (Biochem Soc Trans, 2007). 4. Lee, J. *et al.* Molecular basis for the immunostimulatory activity of guanine nucleoside analogs: Activation of toll-like receptor 7. *Proc. Natl. Acad. Sci. U. S. A.* **100**, 6646–6651 (2003). 5. Heil, F. *et al.* Species-Specific Recognition of Single-Stranded RNA via Till-like Receptor 7 and 8. *Science (80-. ).* **303**, 1526–1529 (2004). 6. Diebold, S. S., Kaisho, T., Hemmi, H., Akira, S. & Reis E Sousa, C. Innate Antiviral Responses by Means of TLR7-Mediated Recognition of Single-Stranded RNA. *Science (80-. ).* **303**, 1529–1531 (2004). 7. Judge, A. D. *et al.* Sequence-dependent stimulation of the mammalian innate immune response by synthetic siRNA. *Nat. Biotechnol.* **23**, 457–462 (2005). 8. Marques, J. T. & Williams, B. R. G. Activation of the mammalian immune system by siRNAs. *Nature Biotechnology* **23**, 1399–1405 (2005). 9. Klinman, D. M., Yi, A. K., Beaucage, S. L., Conover, J. & Krieg, A. M. CpG motifs present in bacterial DNA rapidly induce lymphocytes to secrete interleukin 6, interleukin 12, and interferon γ. *Proc. Natl. Acad. Sci. U. S. A.* **93**, 2879–2883 (1996). 10. Batista-Duharte, A. *et al.* Progress in the use of antisense oligonucleotides for vaccine improvement. *Biomolecules* **10**, (2020). 11. Roberts, W. K., Clemens, M. J. & Kerr, I. M. Interferon induced inhibition of protein synthesis in L cell extracts: An ATP dependent step in the activation of an inhibitor by double stranded RNA. *Proc. Natl. Acad. Sci. U. S. A.* **73**, 3136–3140 (1976). 12. Farrell, P. J. *et al.* Interferon action: Two distinct pathways for inhibition of protein synthesis by double-stranded RNA. *Proc. Natl. Acad. Sci. U. S. A.* **75**, 5893–5897 (1978). 13. Olejniczak, M. *et al.* Sequence-non-specific effects generated by various types of RNA interference triggers. *Biochim. Biophys. Acta - Gene Regul. Mech.* **1859**, 306–314 (2016). 14. Toonen, L. J. A. *et al.* Intracerebroventricular Administration of a 2′-O-Methyl Phosphorothioate Antisense Oligonucleotide Results in Activation of the Innate Immune System in Mouse Brain. *Nucleic Acid Ther.* **28**, 63–73 (2018). 15. Yamada, T., Kawamata, T., Walker, D. G. & McGeer, P. L. Vimentin immunoreactivity in normal and pathological human brain tissue. *Acta Neuropathol.* **84**, 157–162 (1992). 16. Zamanian, J. L. *et al.* Genomic analysis of reactive astrogliosis. *J. Neurosci.* **32**, 6391–6410 (2012). 17. Zakrzewska, A. *et al.* Macrophage-specific gene functions in Spi1-directed innate immunity. *Blood* **116**, (2010). 18. Hornung, V. *et al.* 5′-Triphosphate RNA is the ligand for RIG-I. *Science (80-. ).* **314**, 994–997 (2006). 19. Schlee, M. & Hartmann, G. Discriminating self from non-self in nucleic acid sensing. *Nature Reviews Immunology* **16**, 566–580 (2016). 20. Smith, C. I. E. & Zain, R. Therapeutic Oligonucleotides: State of the Art. *Annu. Rev. Pharmacol. Toxicol.* **59**, 605–630 (2019). 21. Chaturvedi, S. *et al.* Mechanistic exploration of quercetin against metronidazole induced neurotoxicity in rats: Possible role of nitric oxide isoforms and inflammatory cytokines. *Neurotoxicology* **79**, 1–10 (2020). 22. Karamitopoulou, E. *et al.* Active caspase 3 and DNA fragmentation as markers for apoptotic cell death in primary and metastatic liver tumours. *Pathology* **39**, 558–564 (2007). 23. Aubrey, B. J., Kelly, G. L., Janic, A., Herold, M. J. & Strasser, A. How does p53 induce apoptosis and how does this relate to p53-mediated tumour suppression? *Cell Death and Differentiation* **25**, 104–113 (2018).

**Supplementary Table S2. Oligonucleotide sequences**

| Name: | Sequence: | Ta: | Figures: |
|---|---|---|---|
| miR16 | 5’-AGCAGCACGTAAATATTGGC-3’ | +UR, 60°C | 1e; S1d |
| miR21 | 5’-AGCTTATCAGACTGATGTTG-3’ | +UR, 60°C | 1e; S1c, d |
| miR186 | 5’-CAAAGAATTCTCCTTTTGGG-3’ | +UR, 60°C | 1e; S1c, d |
| miR222 | 5’-AGCTACATCTGGCTACTGG-3’ | +UR, 60°C | 1e; S1d |
| miR29a | 5’-TAGCACCATCTGAAATCGG-3’ | +UR, 60°C | S1c |
| miR424 | 5’-CAGCAGCAATTCATGTTTTG-3’ | +UR, 60°C | S1c |
| U6 | 5’-GGATGACACGCAAATTCGTG-3’ | +UR, 60°C | 1e; S1c, d |
| UR (Universal Reverse) | 5’-GTGCAGGGTCCGAGGT-3’ | | 1e; S1c, d; 2d, e; S2d, e |
| SMN2_F | 5’-GGTGTCCACTCCCAGTTCAA-3’ | 60°C | 1f; S1e, f |
| SMN2_R | 5’-GCCTCACCACCGTGCTGG-3’ | 60°C | 1f; S1e, f |
| ATP2A1_F1 | 5’-ACTATCTGGAGGGATAACCACC-3’ | 60°C | S1g |
| ATP2A1_F2 | 5’-ACTATCTGGAGGATCCAGAAGA-3’ | 60°C | S1g |
| ATP2A1_R | 5’-AGCAATCAGCTAGTCAGTTGCC-3’ | 60°C | S1g |
| FMRGFP_F | 5’-GGATCACTCTCGGCATGGA-3’ | +UR, qPCR: 60°C, PCR: 58°C | 2d, e |
| CsACTB_F | 5’-TCCCTGGAGAAGAGCTACGA-3’ | 55°C | 2e |
| CsACTB_R | 5’-AGCACTGTGTTGGCGTACAG-3’ | 55°C | 2e |
| CsFMR1_F | 5’-CCTCTGCTAAACTGATGTTGATGC-3’ | 60°C | S2d, e |
| CsFMR1_R | 5’-TGGTTTTGTACCATTTGGTTGAGT-3’ | 60°C | S2d, e |
| NUB1_F | 5’-TCCCAACCACTTCCTCTCCA-3’ | 60°C | S2d, e |
| NUB1_R | 5’-CATCACTATGCCCCACAGCA-3’ | 60°C | S2d, e |
| CsGAPDH_F | 5’-CACCTTGTCATGTACCATCAATAAA-3’ | +UR, 60°C | 2d, e; S2d, e |
| hFMR1_F | 5’-ATCCCAACAAACCTGCCACA-3’ | 60°C | 3a, d; S3d |
| hFMR1_R | 5’-ATGTGCTCGCTTTGAGGTGA-3’ | 60°C | 3a, d; S3d |
| hFMR1int1_F | 5’-AGAAGATGGAGGAGCTGGTG-3’ | 60°C | 3a |
| hFMR1int1_R | 5’-CCTGAAAAGCACTCAAACTGGA-3’ | 60°C | 3a |
| hFMR1int3_F | 5’-TGTGTCCCCATTGTAAGCAA-3’ | 60°C | S3a |
| hFMR1int3_R | 5’-CTCAACGGGAGATAAGCAG-3’ | 60°C | S3a |
| RNASEH1_F | 5’-CACAGAGGATGAGGCCTG-3’ | 60°C | S3a |
| RNASEH1_R | 5’-CAGTGGCTCACGGAGTC-3’ | 60°C | S3a |
| hGAPDH_F1 | 5’-GAGTCAACGGATTTGGTCGT-3’ | 60°C | 3a; S3a; S6c |
| hGAPDH_R1 | 5’-TTGATTTTGGAGGGATCTCG-3’ | 60°C | 3a; S3a; S6c |
| hGAPDH_F2 | 5’-CACATCGCTCAGACACCATG-3’ | 60°C | 3d; S3d, e; S6c |
| hGAPDH_R2 | 5’-CTTGACGGTGCCATGGAATT-3’ | 60°C | 3d; S3d, e; S6c |
| MALAT_F | 5’-GACGGAGGTTGAGATGAAGC-3’ | 60°C | S3e |
| MALAT_R | 5’-ATTCGGGGCTCTGTAGTCCT-3’ | 60°C | S3e |
| QKI_F | 5’-AGCTGATGGAGCTTGCGATT-3’ | 60°C | S6c + hGAPDH_F2/R2 |
| QKI_R | 5’-AAGGCAAGGGCTGGTGATTT-3’ | 60°C | S6c + hGAPDH_F2/R2 |
| VKORC1L1_F | 5’-CCGGAATCCTGCTCTCCATC-3’ | 60°C | S6c + hGAPDH_F2/R2 |
| VKORC1L1_R | 5’-AATCCTCGACCCCATCTGGA-3’ | 60°C | S6c + hGAPDH_F2/R2 |
| KANSL1Lint3_F | 5’-CCCCTGGCAACTATGTCTGT-3’ | 60°C | S6c + |

| | | | |
|---|---|---|---|
| | | | hGAPDH_F1/R1 |
| KANSL1Lint3_R | 5’-AAGCAACCCAAGTGTCCATC-3’ | 60°C | S6c + hGAPDH_F1/R1 |
| DAZAP1_F | 5’-TCGGTGGAATTCCTCACAAT-3’ | 60°C | S6c + hGAPDH_F1/R1 |
| DAZAP1_R | 5’-CCATGATGTCGTGAAAATGC-3’ | 60°C | S6c + hGAPDH_F1/R1 |
| mP90CGG_F | 5’-ACCTCTCGGGGGCGGGCT-3’ | 58°C | S6a |
| mP90CGG_R | 5’-GGGTCAGCTTGCCGTAGGTGG-3’ | 58°C | S6a |
| mActb_F | 5’-CCAGCCTTCCTTCTTGGGTATG-3’ | 58°C | S6a |
| mActb_R | 5’-AGCTCAGTAACAGTCCGCCT-3’ | 58°C | S6a |
| mGfap_F | 5’-ACTCAATACGAGGCAGTGGC-3’ | 60°C | 5d |
| mGfap_R | 5’-CTCCAGATCGCAGGTCAAGG-3’ | 60°C | 5d |
| mCd68_F | 5’-CCAGCTGTTCACCTTGACCT-3’ | 60°C | 5d |
| mCd68_R | 5’-CAGCAAGAGGGACTGGTCAC-3’ | 60°C | 5d |
| mAif1_F | 5’-CCAAGACCCACCTAGAGCTG-3’ | 60°C | 5d |
| mAif1_R | 5’-GCAGATCTCTTGCCCAGCAT-3’ | 60°C | 5d |
| mTlr3_F | 5’-GCGTTGCGAAGTGAAGAACT-3’ | 60°C | 5d |
| mTlr3_R | 5’-GTTGTTCAAGAGGAGGGCGA-3’ | 60°C | 5d |
| mTlr7_F | 5’-CCCATATCCGTGTACACCGT-3’ | 60°C | 5d |
| mTlr7_R | 5’-AAACCATGTTGGGGGCACAT-3’ | 60°C | 5d |
| mGapdh_F | 5’-ACAGTCCATGCCATCACTGC-3’ | 60°C | 5d |
| mGapdh_R | 5’-GCCTGCTTCACCACCTTCTT-3’ | 60°C | 5d |
| siCtrl | 5'-p-UAAGGCUAUGAAGAGAUACdTdT-3' | | 3a; S3a |
| siCtrl | 5'-p-GUAUCUCUUCAUAGCCUUAdTdT-3' | | 3a; S3a |
| siRNaseH1 | 5'-p-CCGGAAGUUUCAGAAGGGCAUGAAAdTdT-3' | | 3a; S3a |
| siRNaseH1 | 5'-p-UUUCAUGCCCUUCUGAAACUUCCGGdTdT-3' | | 3a; S3a |

Ta, annealing temperature in PCR; dT, deoxythymidine
