# Supplementary material for: Short antisense oligonucleotides alleviate the pleiotropic toxicity of RNA harboring expanded CGG repeats
Source: Nat Commun. 2021 Feb 24;12:1265. doi: 10.1038/s41467-021-21021-w (PMC7904788; doi:10.1038/s41467-021-21021-w)
Supplement: Supplementary file 7 — Description of Additional Supplementary Files [file 41467_2021_21021_MOESM7_ESM.pdf]

Description of Additional Supplementary Files

File Name: Supplementary Data 1

Description: (related to Fig. 5). The datasheet contains the results of differential gene expression analysis of RNA-seq data from striatal tissue of P90CGG mice treated with saline or ASO-CCG and of control mice. Analysis was performed with the use of voom+limma pipeline as described in Material and Methods section. *p*-value was generated using moderated *t*-statistic and adjusted for multiple testing using Benjamini-Hochberg's method (adj.P.Val). Expression levels of presented genes were significantly changed in the saline-treated mice vs the control mice (adj.P.Val <0.05). logFC and adj.P.Val are also provided for other comparisons.

File Name: Supplementary Data 2

Description: (related to Fig. 5). The datasheet contains the results of gene ontology (GO) analysis of genes significantly upregulated and downregulated (AveExpr >1; adj.P.Val <0.05) in the saline vs control and the ASO-CCG vs control groups. Test type was Fisher's Exact with the use of Bonferroni correction for multiple testing. Analyzes based on data of differential gene expression from RNA-seq experiments for striatal tissue of P90CGG mice.

File Name: Supplementary Data 3

Description: (related to Fig. 5). The datasheet contains the results of differential gene expression analysis of RNA-seq data from cortical tissue of P90CGG mice treated with saline or ASO-CCG and of control mice. Analysis was performed with the use of voom+limma pipeline as described in Material and Methods section. *p*-value was generated using moderated *t*-statistic and adjusted for multiple testing using Benjamini-Hochberg's method (adj.P.Val). The presented genes contain repeat tracts of at least 6 CGGs in their mRNA sequences.

File Name: Supplementary Data 4

Description: (related to Fig. 5). The datasheet contains the results of differential gene expression analysis of RNA-seq data from striatal tissue of P90CGG mice treated with saline or ASO-CCG and of control mice. Analysis was performed with the use of voom+limma pipeline as described in Material and Methods section. *p*-value was generated using moderated *t*-statistic and adjusted for multiple testing using Benjamini-Hochberg's method (adj.P.Val). The presented genes contain repeat tracts of at least 6 CGGs in their mRNA sequences in their mRNA sequences.
